# Prevalence and Impact of Food Allergies Among Jordanian Schoolchildren: A Comprehensive Analysis of Parent-Reported Data and Associated Atopic Conditions

**DOI:** 10.1155/ijpe/8255384

**Published:** 2025-04-16

**Authors:** Nour Yousef, Raghda O'leimat, Mohammad Abdelraheem, Eyad Altamimi

**Affiliations:** ^1^Faculty of Medicine, Jordan University of Science and Technology, Irbid, Jordan; ^2^Pediatric Department, Faculty of Medicine, Jordan University of Science and Technology, Irbid, Jordan

**Keywords:** allergens, allergy, anaphylaxis, atopy, food

## Abstract

**Background:** Food allergies, characterized by immune reactions to food proteins, have emerged as an increasing global concern, impacting over 10% of the population. This study investigates the prevalence of food allergies among schoolchildren aged 5–14 years in northern and central Jordan.

**Methods:** A randomized cross-sectional online questionnaire was administered to students aged 5–14 years residing in the central and northern regions of Jordan. Parents of these students (*n* = 1629) completed the questionnaire, which comprised information on demographics, dietary habits, food allergies, and allergy history.

**Results:** The parent-reported food allergies exhibited a prevalence of 11.5% (95% confidence interval = 10–13%). Of these reported allergies, 10.1% (95% CI = 8.6–11.7%) were classified as convincing, while only 5.7% (95% CI = 4.5–6.8%) received a formal diagnosis from a healthcare professional. The most commonly reported allergens included eggs (17.8%), cow's milk (15.8%), peanuts (14.5%), and strawberries (13.8%). The majority of children (67.1%) had a single reported allergen, while 15.8% had two, and 17.2% had more than two allergens identified. Food allergies were significantly more prevalent among children with comorbid atopic conditions and those with a family history of allergies. Additionally, regional variations were observed, with high prevalence rates in metropolitan regions.

**Conclusion:** This study emphasizes the significance of considering persuasive food allergy (FA) data in addition to parent-reported information. The findings highlight the considerable burden of food allergies and their relationship with other atopic disorders. Comprehensive management strategies and further research to elucidate the underlying causes of food allergies are necessary.

Summary

• Food allergies affect nearly one out of 10 schoolchildren in Jordan, with egg and milk allergies emerging as the most prevalent types. Our study also reveals a notable correlation between food allergies and coexisting atopic conditions, as well as a familial predisposition to allergic reactions.
• The management of food allergies poses a considerable challenge to the healthcare sector, necessitating comprehensive strategies for prevention and treatment. It is imperative to implement proactive measures to address the growing burden of food allergies among children, ensuring their well-being and quality of life.

## 1. Introduction

Food allergies are an abnormal immune response triggered by the consumption of certain foods or components known as allergens. These reactions can cause symptoms ranging from moderate to severe and affect various organ systems, including the skin, gastrointestinal tract, respiratory system, and cardiovascular system [1]. The global prevalence of food allergies has significantly increased in recent years, leading to health challenges and tight dietary management [2–4].

Schoolchildren are particularly vulnerable due to their developing immune systems and limited exposure to a variety of foods. Food allergies can disrupt daily routines, limit nutrition, social contacts, and overall quality of life. The search for suitable dietary replacements becomes difficult, especially in school settings where allergy exposure remains a concern. Food allergies place an overwhelming financial burden on both families and the government due to the high costs of medical care, special diets, and emergency treatments [1, 5, 6].

The double-blind placebo-controlled food challenge (DBPCFC) is the gold standard for food allergy diagnosis, but due to the high risks and expenses involved, some studies use questionnaires to determine the prevalence of food allergies [7–9].

Food allergies have been associated primarily with wealthy countries, but current evidence suggests that prevalence is increasing in underdeveloped countries as well. This growth could be related to a more westernized lifestyle or a lack of rigorous research into food allergies in developing countries. Rates as high as 16.1% were observed in Saudi Arabia, with eggs being the most common cause (4.9%) [10]. In Lebanon, 6% of 2610 schoolchildren aged 3–17 had food allergies, similar to percentages recorded in the United States. The most common allergies are cow's milk and milk products (22.46%), fruits and vegetables (16.58%), eggs (8.02%), and nuts (5.88%) [11].

Despite the increase in food allergy prevalence, the topic remains overlooked in some areas, raising concerns about children's safety and the possibility of death. The current study aims to investigate the prevalence of parent-reported food allergies among Jordanian schoolchildren aged 5–14 years and evaluate the degree of association between food allergies and comorbidities, such as asthma and atopic dermatitis.

## 2. Methods

This study aimed to evaluate the prevalence of food allergies among schoolchildren in Jordan's central and northern regions, as well as investigate any correlations with other atopic diseases. Moreover, it investigated the influence of early life factors, such as the kind and timing of weaning meals, on the development of food allergies in this population.

### 2.1. Definitions

Convincing food allergy refers to cases where the symptoms reported by parents are consistent with medically recognized allergic reactions to food, particularly those that involve immune system responses such as hives, swelling, difficulty breathing, or anaphylaxis [12].

A severe allergic reaction was defined as a parent report of at least two stringent symptoms (hives, rash, swelling [except lip or tongue], lip and/or tongue swelling, difficulty swallowing, throat tightening, chest tightening, trouble breathing, wheezing, vomiting, chest pain, tachycardia, fainting, dizziness, feeling light headed, or low blood pressure) from two different body systems during a child's most severe reaction to a given food [12].

Early introduction of solid food (weaning) occurs when solid food is introduced before the age of 4 months.

Appropriate introduction of solid food (weaning) occurs when solid food is introduced between 4 and 6 months.

Late introduction of solid food (weaning) occurs when solid food is introduced after the age of 7 months.

Early introduction of cow's milk occurs when cow's milk is introduced before the age of 12 months.

Appropriate introduction of cow's milk occurs when cow's milk is introduced after 12 months of age.

### 2.2. Study Design and Sample

To guarantee comprehensive representation across the governorate's geographical regions and educational sectors, schools were chosen at random. Grades 1 through 8 at each designated school were sampled for questionnaire delivery to families. Between October and November 2023, 24 schools (13 public and 11 private) were picked from three governorates (Amman, Irbid, and Al-Mafraq). The school allocation was as follows: 2425 children from four private and four public schools in Amman, 2000 students from five private and four public schools in Irbid, and 1788 pupils from two private and five public schools in Al-Mafraq. Amman had 269,865 children aged 5–14, Irbid had 199,263, and Al-Mafraq had 12,080. The sample size was calculated using Cohran's sample size calculation to provide a 95% confidence level, with a minimum of 384, 384, and 373 pupils from Amman, Irbid, and Al-Mafraq, respectively.

### 2.3. Data Collection

Data was collected using a previously validated research-specific online questionnaire created which was adopted from a previous study in Jordan [13] and supplemented with content validation feedback from a consultant pediatric gastroenterologist (EA). The Jordanian Ministry of Education granted permission for research participation and the use of child health information. The questionnaire was in Arabic and verified by noncontent experts, after which 50 randomly selected participants judged its clarity and understandability (responses were not used in the final data analysis). The questionnaire in a google form was posted at the parent–teacher groups. The questionnaire which includes demographic information, eating history, food allergy specifics, and atopic history for both children and their families was rigorously designed for clarity and conciseness. School administrators ensured questionnaire clarity and time efficiency, screening replies, and eliminating duplicates. Managers sent out monthly reminders to parent–teacher groups to improve response rates. A plateau was observed after 2 months, indicating adequate response levels. To protect data integrity, a restriction was imposed that allowed only one reply per account.

### 2.4. Analysis and Statistics

Reported allergies were classified according to symptom severity, using strict criteria defined by the expert opinion's panel [12].

Allergies were considered convincing if they demonstrated at least one of the severe symptoms (hives, rash, swelling [except lip or tongue], lip and/or tongue swelling, difficulty swallowing, throat tightening, chest tightening, trouble breathing, wheezing, vomiting, chest pain, tachycardia, fainting, dizziness, feeling light headed, or low blood pressure), with severity differentiated further by symptom multiplicity and organ system involvement. The introduction of weaning food was grouped by age. The statistical analysis used Pearson's chi-squared or Fisher's exact tests for nominal categorical variables and the Kruskal–Wallis test for ordinal data. The analysis was conducted using SPSS Version 26.0.0, and the statistical significance was set at two-tailed *p*-values of < 0.05.

### 2.5. Ethics Statement

The KAUH Institutional Review Board (IRB) and research committee approved the study's ethical conduct, under the approval number (20230450). The study is carried out in compliance with the ethical guidelines in place at our institute, taking the Helsinki Declaration as an ethical guideline for research involving human subjects. Informed consent was obtained from the parents/guardians of all participants.

## 3. Results

The response rate to our questionnaire was 26.2%, and 1629 parents completed it. The study excluded 123 people who declined to participate and four due to missing data. The final analytical sample consisted of 1502 schoolchildren, 768 females and 734 males, spread across three geographical areas: 32.6% in Amman, 39.9% in Irbid, and 27.4% in Al-Mafraq. The average age of the children was 9.2 ± 2.419 years. 47.6% attended public school, whereas 52.4% went to private school (Table 1).

About 31.8% of children reported having atopic disorders, including 12.5% with asthma, 6.1% with eczema, 16.4% with allergic rhinitis, 6.1% with allergic conjunctivitis, and 1.1% with poor growth (Table 2). Additionally, 47.3% of children had a positive family history of atopic illnesses, whereas 6.7% had a family history of food allergy (Table 1).

According to family accounts, the prevalence of food allergies was 11.5% (95% confidence interval = 10–13%). Among these, 10.1% (95% CI = 8.6–11.7%) claimed to have a persuasive food allergy, whereas 5.7% (95% CI = 4.5–6.8%) were diagnosed by a doctor. The majority (67.1%) of children with compelling food allergies reported allergies to a single allergen, followed by 15.8% to two allergens and 17.2% to more than two allergens.

Egg allergy was the most common allergen recorded, affecting around 17.8% of children with compelling allergies, followed by cow's milk (15.8%), peanuts (14.5%), and strawberries (13.8%). Sesame and hazelnut allergies were confirmed in all instances (100%), followed by peanuts in 95.7% and strawberries in 95.5% (Table 3).

Considering severity of reactions, cow's milk and eggs were the most common, followed by peanuts. Furthermore, pistachio was the allergen with the largest proportion of severe reactions, accounting for 88.9% of cases, followed by sesame (76.9%) and fish (58.8%). In contrast, strawberries, kiwi, and wheat had the highest frequency of mild to moderate allergies (Table 3).

Regarding emergency room visits, allergy due to pistachio was highest, with 77.8% of pistachio-allergy cases requiring a visit to the emergency department. Furthermore, sesame, as well as cow's milk and eggs, had the highest frequency of severe allergies requiring an emergency department visit (Table 3).

The research found no statistically significant difference in reported food allergies between males and females (*p* = 0.419) (Table 1). Similarly, no significant difference was observed in the mean age between children with and without documented food allergies (*p* = 0.425). Surprisingly, no statistically significant difference was found in average age between children who had overcome their food allergies and those who continued to have them.

Regarding regional variance, the prevalence of reported food allergies was highest in Amman (14.5%), followed by Irbid (10.8%), and the lowest in Al-Mafraq (9.0%) (*p* = 0.028). Furthermore, parents of children in private schools reported a greater incidence of food allergies (13.2%) than those in public schools (9.7%), with the difference being statistically significant (*p* = 0.035) (Table 1).

Examining early life characteristics, we discovered that weaning age, method of delivery, feeding type (breastfeeding or formula), breastfeeding length, and age of cow milk introduction had no statistical relationship with reported, convincing, or diagnosed food allergies. However, birth order was found to be a significant influencer, with only children having the highest frequency of reported food allergies (17.2%), followed by eldest children (15.4%), youngest children (11.5%), and finally middle children (8.3%). Surprisingly, no statistically significant link was observed between smoking and any measure of allergy, whether reported, compelling, or diagnosed (Table 4).

Children with a history of atopic illnesses (e.g., allergic rhinitis, asthma, allergic eczema, or allergic conjunctivitis) had significantly higher rates of reported, convincing, and diagnosed food allergies than those without food allergy (*p* ≤ 0.0011). However, a history of poor growth was only strongly associated with a physician-diagnosed food allergy (Table 2). Similarly, a family history of atopy and food allergies was a strong predictor of reported food allergies. Having a mother with an allergic disease (*p* ≤ 0.0011) but not a father was connected with an increased incidence of food allergy in the offspring (*p* ≤ 0.0011). Having family members with allergies increased the chance of food allergy (*p* ≤ 0.0011).

**Figure 1:**
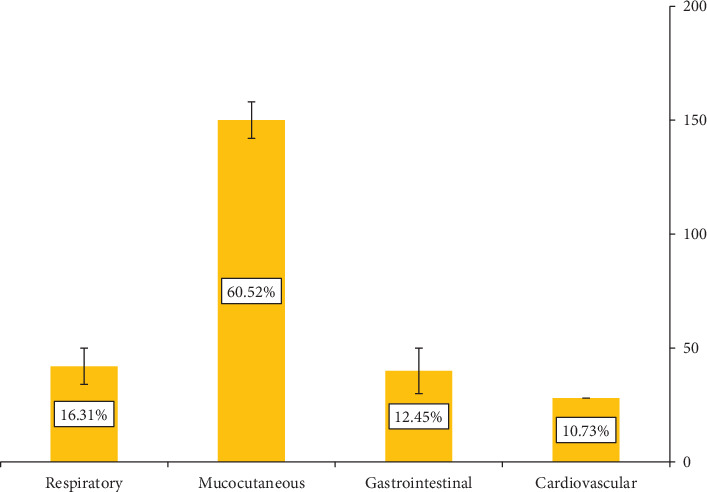
Reported system involvement in patients' with reported food allergy.

Mucocutaneous involvement was the most often reported system impacted by food allergies, accounting for 60.52% of children reporting food allergies (Figure 1), with skin rash being the most common symptom (48.6%). The analysis indicated no statistically significant variations in system involvement or symptom prevalence among the three governorates evaluated.

## 4. Discussion

The study examined the prevalence of food allergies in children using parent-reported data. Results showed that 11.5% of parents reported a food allergy in their children, while 5.7% had a physician-diagnosed allergy. This rate is consistent with a previous study in Al-Karak, Jordan. However, the study found a higher prevalence of physician-diagnosed food allergies (5.7% vs. 3.4%), possibly due to the larger geographical area [13]. A Kuwait study reported an even higher frequency of physician-diagnosed food allergies at 8.2% [14].

The study focuses on estimating the prevalence of compelling food allergies in Jordan, using rigorous symptoms rather than participant reports. The study found a negligible difference in percentages between reported and convincing allergies (11.5% vs. 10.1%), indicating Jordanians' strong understanding of food allergies. This differs from a US study that found a 7.6% prevalence of compelling food allergies [12].

Parent-reported food allergy prevalence varies across countries. Nearby Arab countries, such as Kuwait, Bahrain, and Saudi Arabia, report high rates of 12.7%, 15.5%, and 16.1%, respectively [10, 14, 15], while the UAE shows a lower rate of 8.0% [16]. Global comparisons also reveal wide variations in parent-reported rates, with Italy and the United States having rates of 10.5% and 11.4%, respectively [12, 17].

Factors contributing to this diversity include dietary choices, environmental conditions, cultural behaviors, genetic predisposition, study design, demographics, methodology, and research criteria. Further research is needed to understand these discrepancies.

Our study found that eggs, cow's milk, peanuts, and strawberries are the most commonly reported dietary allergens. However, the sequence of specific allergies varies by country. For instance, cow's milk is the most prevalent allergy in Italy followed by eggs [17], peanuts in the United States [18], and shrimp in Mexico [19].

Recent research [13–16] indicates a higher prevalence of food allergies in children with concurrent atopic illnesses like allergic rhinitis, asthma, eczema, and allergic conjunctivitis. Children with these conditions are more likely to develop food allergies compared to healthy children. However, food allergies are not significantly associated with asthma [10], although exposure to food allergens can lead to precipitate asthma and allergic rhinitis [20].

A single family member with a history of allergic disease increases the child's chance of food allergies by 1.4-fold, while having two or more family members increases the risk by 1.8-fold [21]. A previous Japanese study found a significant positive connection between all allergic disorders of either or both parents and their child's food allergy [22].

These findings are consistent with our study, which found a robust link between a family history of allergic disease and the risk of food allergy in children, with a notable maternal predominance impact and an increased risk of food allergy when more than one relative is affected.

Food allergy prevalence in Jordan is highest in urban areas like Amman, followed by rural areas like Irbid, and lowest in nomadic areas like Al-Mafraq. This contrasts with previous research in South Africa, the United States, and China [23–25].

Toskala et al. suggest that urban areas often experience higher pollution levels, which may increase the risk of developing atopy and asthma. However, it remains unclear whether this increased risk extends to food allergies [26].

In contrast, the hygiene hypothesis, proposed by David P. Strachan, suggested that the rise in allergies and autoimmune diseases in urban areas is linked to reduced exposure to infectious agents, parasites, and microbes during early childhood. In addition to other factors such as smaller family sizes, increased use of antibiotics, and modern lifestyle changes in urban communities [27].

Mucocutaneous involvement was the most often reported system impacted by food allergies, accounting for 60.52% of children, with a skin rash being the most common symptom (48.6%). This is consistent with international rates observed in China [28], Europe [29], bordering Lebanon [11], Saudi Arabia [10], and Kuwait [14].

The relationship between smoking exposure and food allergies is controversial, but our findings show no link. Studies have found a link between food allergies and secondhand smoke, but the causes are unknown. Children whose parents smoked during their early months of life have a greater chance of sensitization [30, 31]. This discrepancy with our findings might be attributed to the cultural practice of avoiding smoking in front of children during their first few months of life. Early life influences on food allergies are conflicting, with some studies showing no link, while others suggest a protective effect or increased incidence [32, 33].

The study examined the link between food allergy prevalence and early life characteristics like weaning age, delivery method, feeding type, breastfeeding length, and cow milk introduction age. Results showed no significant association with food allergies.

However, one Bahraini study found that formula-fed children were more likely to have food allergies [15]. Our study found that the order of birth significantly affects food allergies, with the eldest and only children being more likely to develop them. This finding aligns with previous research, including Kuwait's study [14] and a Japanese survey [34].

The study has limitations, including a lack of objective data due to reliance on parental reports, which raises the possibility of recollection bias. To address this, the study calculated food allergy rates based on reported symptoms rather than parental diagnosis. The cross-sectional questionnaire-based technique had a low response rate, heightened by the sensitivity to response bias. However, the prevalence of food allergies (11.5%) reported is consistent with a prior study in Al-Karak, Jordan, with a higher response rate (88.6%).

The study evaluated the incidence of food allergies among Jordanian schoolchildren, examining their relationship with atopic disease and identifying risk factors. It found a similar incidence of parent-reported allergies to those in bordering and Western countries. The study is the first in the Middle East to investigate the association between food allergy and compelling allergy, contributing to the scarcity of such research. It discovered a direct link between food allergies and other atopic disorders like asthma and allergic rhinitis, and their coexistence was significant in personal and family medical histories.

## Figures and Tables

**Table 1:** Patients demographics and prevalence of food allergy distribution.

	**All children**	**Parent-reported FA**	**Convincing FA**	**Physician-diagnosed FA**
*Governorate*				
Amman	490 (32.6)	71 (14.5)	63 (12.9)	38 (7.8)
Irbid	600 (39.9)	65 (10.8)	56 (9.3)	29 (4.8)
Al-Mafraq	412 (27.4)	37 (9.0)	33 (8.0)	18 (4.4)
*p*-value		0.028	0.040	0.048
*Gender*				
Male	734 (48.9)	90 (12.3)	77 (10.5)	47 (6.4)
Female	768 (51.1)	83 (10.8)	75 (9.8)	38 (4.9)
*p*-value		0.419	0.669	0.264
*Type of school*				
Public	715 (47.6)	69 (9.7)	60 (8.4)	33 (4.6)
Private	787 (52.4)	104 (13.2)	92 (11.7)	52 (6.6)
*p*-value		0.035	0.040	0.117
*Atopy history*				
Positive	477 (31.8)	87 (18.2)	80 (16.8)	49 (10.3)
Negative	1025 (68.2)	86 (8.4)	72 (7.0)	36 (3.5)
*p*-value		≤ 0.0011	≤ 0.0011	≤ 0.0011
*Family atopy history*				
Positive	710 (47.3)	116 (16.3)	105 (14.8)	55 (7.7)
Negative	792 (52.7)	57 (7.2)	47 (5.9)	30 (3.8)
*p*-value		≤ 0.0011	≤ 0.0011	≤ 0.0011
*Family food allergy history*				
Positive	101 (6.7)	38 (37.6)	33 (32.7)	21 (20.8)
Negative	1401 (93.3)	135 (9.6)	119 (8.5)	64 (4.6)
*p*-value		≤ 0.001	≤ 0.001	< 0.001

**Table 2:** Prevalence of atopic disorders in children reported to have different forms of food allergies.

	**All children**	**Parent-reported FA**	**Convincing FA**	**Physician-diagnosed FA**
*Asthma*				
Positive	187 (12.5)	37 (21.4)	34 (22.4)	21 (24.7)
Negative	1315 (87.5)	136 (78.6)	118 (77.6)	64 (75.3)
*p*-value		≤ 0.0011	≤ 0.0011	≤ 0.0011
*Eczema*				
Positive	92 (6.1)	34 (19.7)	32 (21.1)	(25.9)22
Negative	1410 (93.9)	139 (80.3)	120 (78.9)	63 (74.1)
*p*-value		≤ 0.0011	≤ 0.0011	≤ 0.0011
*Allergic rhinitis*				
Positive	246 (16.4)	40 (23.1)	36 (23.7)	21 (24.7)
Negative	1256 (83.6)	133 (76.9)	116 (76.3)	64 (75.3)
*p*-value		0.016	0.015	0.048
*Allergic conjunctivitis*				
Positive	91 (6.1)	23 (13.3)	21 (13.8)	16 (18.8)
Negative	1411 (93.9)	150 (86.7)	131 (86.2)	69 (81.2)
*p*-value		≤ 0.0011	≤ 0.0011	≤ 0.0011
*Poor growth*				
Positive	17 (1.1)	4 (2.3)	4 (2.6)	4 (4.7)
Negative	1485 (98.9)	169 (97.7)	148 (97.4)	81 (95.3)
*p*-value		0.122	0.084	0.013

**Table 3:** Distribution and severity of Food allergy in Jordanian Children.

	**Eggs** **N** = 30	**Cow's milk** **N** = 29	**Peanuts** **N** = 23	**Strawberry** **N** = 22	**Fish** **N** = 17	**Sesame** **N** = 13	**Kiwi** **N** = 12	**Wheat** **N** = 12	**Hazelnut** **N** = 12	**Pistachio** **N** = 9
*Severe*	13 (43.3)	15 (51.7)	12 (52.2)	5 (22.7)	10 (58.8)	10 (76.9)	4 (33.3)	5 (41.7)	7 (58.3)	8 (88.9)
*Mild to moderate allergy*	17 (56.7)	14 (48.3)	11 (47.8)	17 (77.3)	7 (41.2)	3 (23.1)	8 (66.7)	7 (58.3)	5 (41.7)	1 (11.1)
*Convincing FA*	27 (90.0)	24 (82.8)	22 (95.7)	21 (95.5)	16 (94.1)	13 (100.0)	9 (75.0)	11 (91.7)	12 (100.0)	8 (88.9)
*ER visit*	18 (60.0)	22 (75.9)	14 (60.9)	10 (45.5)	12 (70.6)	10 (76.9)	4 (33.3)	7 (58.3)	4 (33.3)	7 (77.8)

**Table 4:** Early life events and environmental factors in relation to reported food allergy.

**Column label**	**All children**	**Parent-reported FA**	**Convincing FA**	**Physician-diagnosed FA**
*Weaning age*				
Early	116 (7.7)	13 (11.2)	9 (7.8)	10 (8.6)
Appropriate	915 (61.1)	115 (12.6)	102 (11.1)	57 (6.2)
Late	466 (31.1)	44 (9.4)	40 (8.6)	18 (3.9)
*p*-value		0.226	0.224	0.072
*Age of cow milk introduction*				
Early	625 (41.6)	66 (10.6)	62 (9.9)	34 (5.4)
Appropriate	872 (58.1)	106 (12.2)	89 (10.2)	51 (5.8)
*p*-value		0.366	0.931	0.821
*Breastfeeding*				
Natural	524 (34.9)	62 (11.8)	56 (10.7)	30 (5.7)
Formula	278 (18.5)	30 (10.8)	25 (9.0)	15 (5.4)
Mixed	700 (46.6)	81 (11.6)	71 (10.1)	40 (5.7)
*p*-value		0.906	0.751	0.978
*Mode of delivery*				
Vaginal	969 (64.5)	105 (10.8)	91 (9.4)	49 (5.1)
C-section	533 (35.5)	68 (12.8)	61 (11.4)	36 (6.8)
*p*-value		0.273	0.212	0.199
*Child order*				
Eldest	442 (29.4)	68 (15.4)	61 (13.8)	31 (7.0)
Middle	555 (37)	46 (8.3)	42 (7.6)	22 (4.0)
Youngest	470 (31.3)	54 (11.5)	44 (9.4)	32 (6.8)
Only child	29 (1.9)	5 (17.2)	5 (17.2)	0 (0.0)
*p*-value		0.008	0.011	0.100
*Exposure to smoking*				
Yes	860 (57.3)	95 (11.0)	87 (10.1)	47 (5.5)
No	642 (42.7)	78 (12.1)	65 (10.1)	38 (5.9)
*p*-value		0.514	1.000	0.736

## Data Availability

The data that support the findings of this study are available on request from the corresponding author. The data are not publicly available due to privacy or ethical restrictions.

## Nomenclature

FA: food allergy
